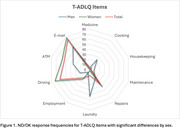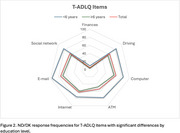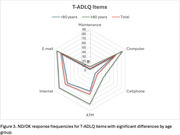# Assessing Functional Ability in Cognitive Aging: Challenges and Sociocultural Factors

**DOI:** 10.1002/alz70857_106757

**Published:** 2025-12-25

**Authors:** Teresa Parrao, Rodrigo Saguez, Daniela Thumala, Patricia Lillo, Roque Villagra, Andrea Slachevsky

**Affiliations:** ^1^ Universidad Alberto Hurtado, Santiago, Chile; ^2^ Geroscience Center for Brain Health and Metabolism (GERO), Santiago, Chile; ^3^ Neuropsychology and Clinical Neuroscience Laboratory (LANNEC), Physiopathology Department – Institute of Biomedical Sciences (ICBM), Neuroscience and East Neuroscience Departments, Faculty of Medicine, Universidad de Chile, Santiago, Chile, Santiago, Chile; ^4^ Department of Psychology, University of Chile, Santiago, Chile; ^5^ Geroscience Center for Brain Health and Metabolism (GERO), Santiago, Metropolitana, Chile; ^6^ East Neuroscience Departments, Faculty of Medicine, University of Chile, Santiago, Chile; ^7^ East Neurology Department, University of Chile, Santiago, Chile; ^8^ Neurology Department, Hospital del Salvador, University of Chile, Santiago, Región Metropolitana de Santiago, Chile; ^9^ Memory and Neuropsychiatric Clinic (CMYN), Neurology Service, Hospital del Salvador and Faculty of Medicine, Universidad de Chile, Santiago, Chile; ^10^ Neuropsychology and Clinical Neuroscience Laboratory (LANNEC), Physiopathology Department ‐ ICBM, Neuroscience and East Neuroscience Departments, Faculty of Medicine, University of Chile, Santiago, Chile

## Abstract

**Background:**

Functional ability is a key indicator of cognitive health, distinguishing cognitively unimpaired (CU) aging, mild cognitive impairment (MCI), and dementia. However, sociocultural factors such as gender roles, education, and age‐related differences influence activity participation, complicating assessments. The Activities of Daily Living Questionnaire‐Technologies (T‐ADLQ) evaluates three domains of functional ability: instrumental activities of daily living (iADL), basic activities of daily living (bADL), and advanced activities of daily living (aADL). It includes a response option, “Never did this activity/Don't know” (ND/DK), which may reflect disparities unrelated to cognitive impairment.

**Method:**

This study analyzed ND/DK responses in the GERO cohort, a prospective study in Santiago, Chile, following 291 dementia‐free older adults with cognitive complaints (≥70 years) for three years to assess cognitive, functional, psychosocial, and medical factors. We examined how gender, education, and age influenced functional ability assessments using a test for equality of proportions to compare ND/DK response distributions.

**Result:**

Men were significantly more likely to report never engaging in household tasks such as taking pills (4.1% vs. 0.5%, *p* = 0.0049), cooking (24.5% vs. 1.0%, *p* <0.001), housekeeping (10.2% vs. 1.0%, *p* = 0.0004), and laundry (53.1% vs. 1.9%, *p* <0.001). Conversely, women had higher ND/DK responses in home repairs (42.0% vs. 19.1%, *p* = 0.0029), employment (23.2% vs. 6.1%, *p* = 0.0071), driving (72.5% vs. 20.4%, *p* <0.001), and ATM usage (58.0% vs. 34.7%, *p* = 0.0033). Participants with <6 years of education had significantly higher ND/DK responses in financial management (9.4% vs. 3.0%, *p* = 0.0393), driving (83.0% vs. 56.9%, *p* = 0.0003), and technology use (computer access: 84.9% vs. 61.4%, *p* = 0.0013; email access: 96.2% vs. 68.8%, *p* <0.001). Individuals aged ≥80 showed higher ND/DK rates in home maintenance (7.7% vs. 1.6%, *p* = 0.0147), mobile phone use (33.9% vs. 13.7%, *p* = 0.0003), and internet access (78.5% vs. 57.4%, *p* = 0.0024).

**Conclusion:**

Sociocultural factors strongly influence functional ability assessments, with notable disparities by gender, education, and age. These findings highlight the need to interpret ND/DK responses carefully to prevent misclassification of functional impairment in cognitive aging evaluations. Additionally, improving assessment tools to account for sociocultural variations is essential for more accurate evaluations.